# Sebaceous carcinoma of the breast predominantly characterized by intraductal growth: a case report

**DOI:** 10.1186/s40792-020-0799-y

**Published:** 2020-02-24

**Authors:** Koichi Ohno, Toshihiro Okada, Toshitsugu Nakamura, Hiroshi Koyama

**Affiliations:** 1 Department of Breast and Endocrine Surgery, Suwa Red Cross Hospital, 5-11-50, Kogan-douri, Suwa, Nagano, 392-8510 Japan; 2 Department of Diagnostic Pathology, Suwa Red Cross Hospital, 5-11-50, Kogan-douri, Suwa, Nagano, 392-8510 Japan; 3 Koyama Clinic, 1-2557-1, Jonan, Suwa, Nagano, 392-0017 Japan

**Keywords:** Breast carcinoma, Sebaceous carcinoma, Carcinoma in situ, Immunohistochemistry

## Abstract

**Background:**

Sebaceous carcinoma (SC) is frequently classified as periocular or extraocular. Extraocular SC is rare and mainly occurs in the head and neck, the major salivary glands, or oral mucosa. SC of the breast, lung, and ovary is particularly rare, and the few cases of SC of the breast predominantly exhibit intraductal growth.

**Case presentation:**

A 47-year-old Japanese woman was referred to our hospital with accumulated polymorphic calcification in the left breast which was detected using mammography. Ultrasonography revealed an irregular 13-mm mass in the left breast, and analysis of a core needle biopsy revealed noninvasive ductal carcinoma. Total mastectomy and sentinel lymph node biopsy were performed. Histopathology demonstrated that carcinoma in situ (CIS) represented a significant lesion, and the cytoplasm of tumor cells was clear with numerous minute vacuoles. Immunohistochemical analysis demonstrated that most tumor cells expressed adipophilin. Together, these findings led to a diagnosis of SC, mainly comprising CIS.

**Conclusions:**

We encountered a rare case of SC of the breast with predominant CIS.

## Background

Sebaceous carcinoma (SC) is frequently classified as periocular or extraocular [1]. Extraocular SC is rare, occurring mainly in the head and neck, major salivary glands, oral mucosa, breasts, lungs, and ovaries [2–6]. Although carcinoma in situ (CIS) lesions of SC are rare, particularly associated with extraocular SC, they occur in the head, neck, and upper arm [7–10]. We are unaware of reports of SC of the breast with predominant CIS. For the first time to our knowledge, we report here a case of SC of the breast predominantly characterized by intraductal growth and review the relevant literature.

## Case presentation

**Fig. 1:**
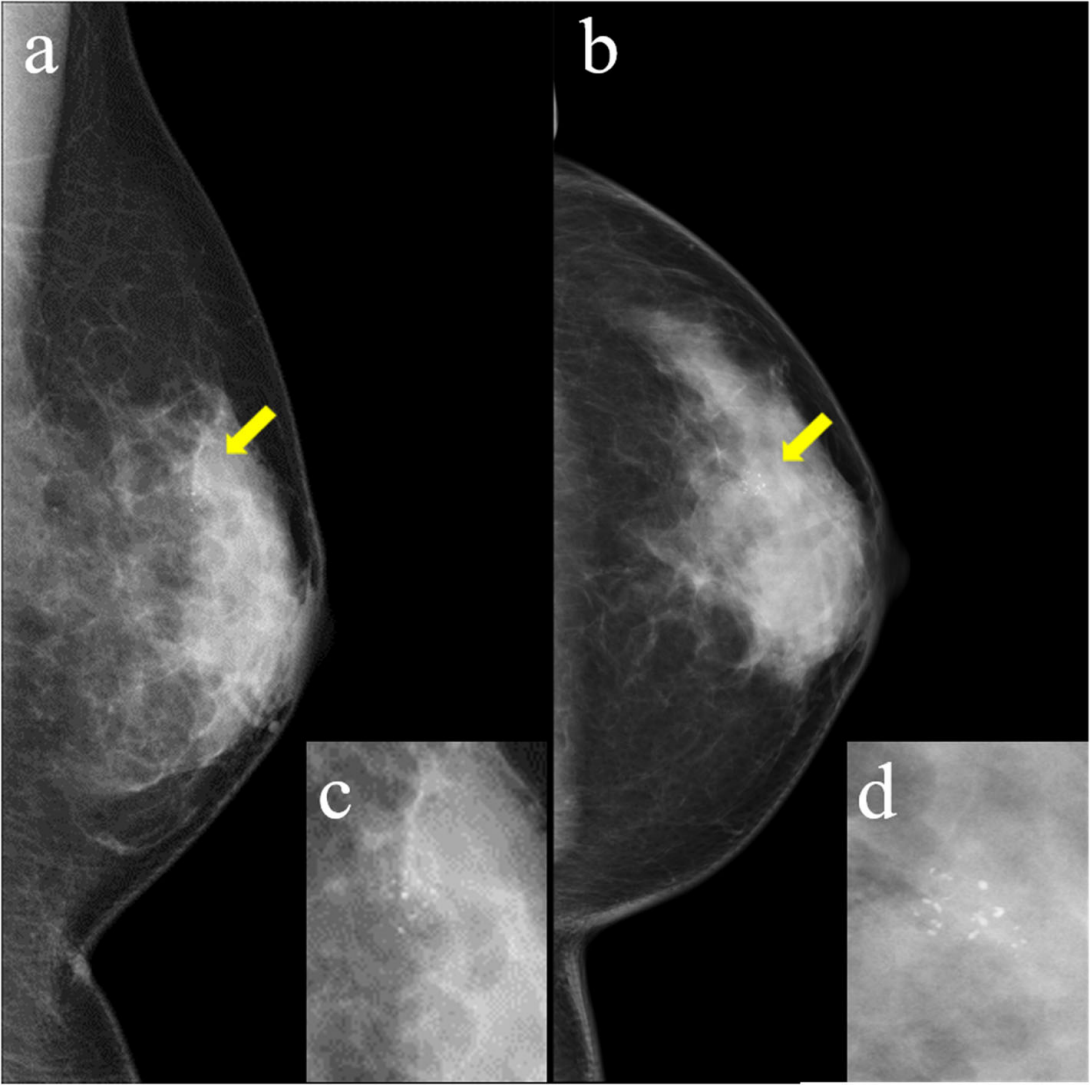
Mammogram. Mammogram showing clustered pleomorphic calcification in the left MLO-M area (**a**) and CC-O area (**b**). **c**, **d** Enlargements of the areas indicated by yellow arrows

**Fig. 2:**
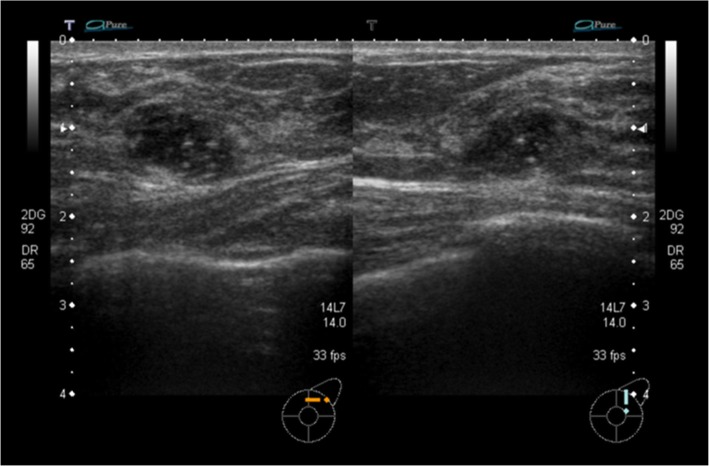
Ultrasonography. Ultrasonography showing a hypoechoic irregular mass (13 mm × 12 mm × 7 mm) with clear roughening of the boundary and a high echo spot inside in the left CD area

**Fig. 3:**
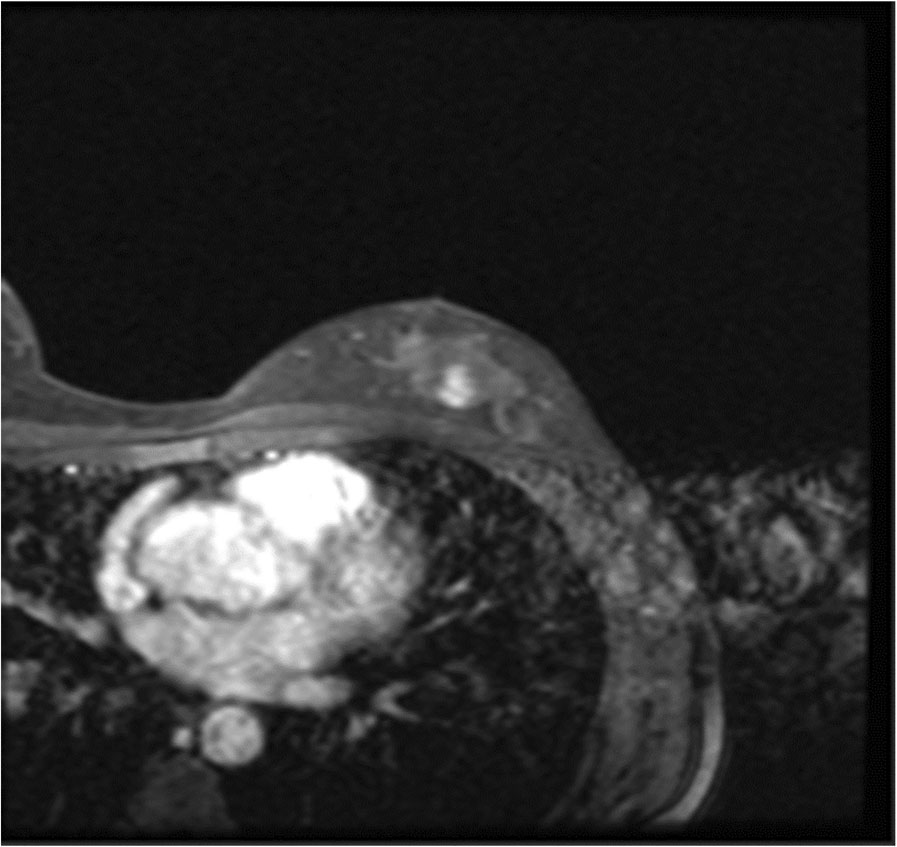
Contrast-enhanced magnetic resonance imaging (CE-MRI). CE-MRI showing an irregular mass with early arterial enhancement (14 mm × 11 mm × 12 mm) that was confined to the same area in the left C area

**Fig. 4:**
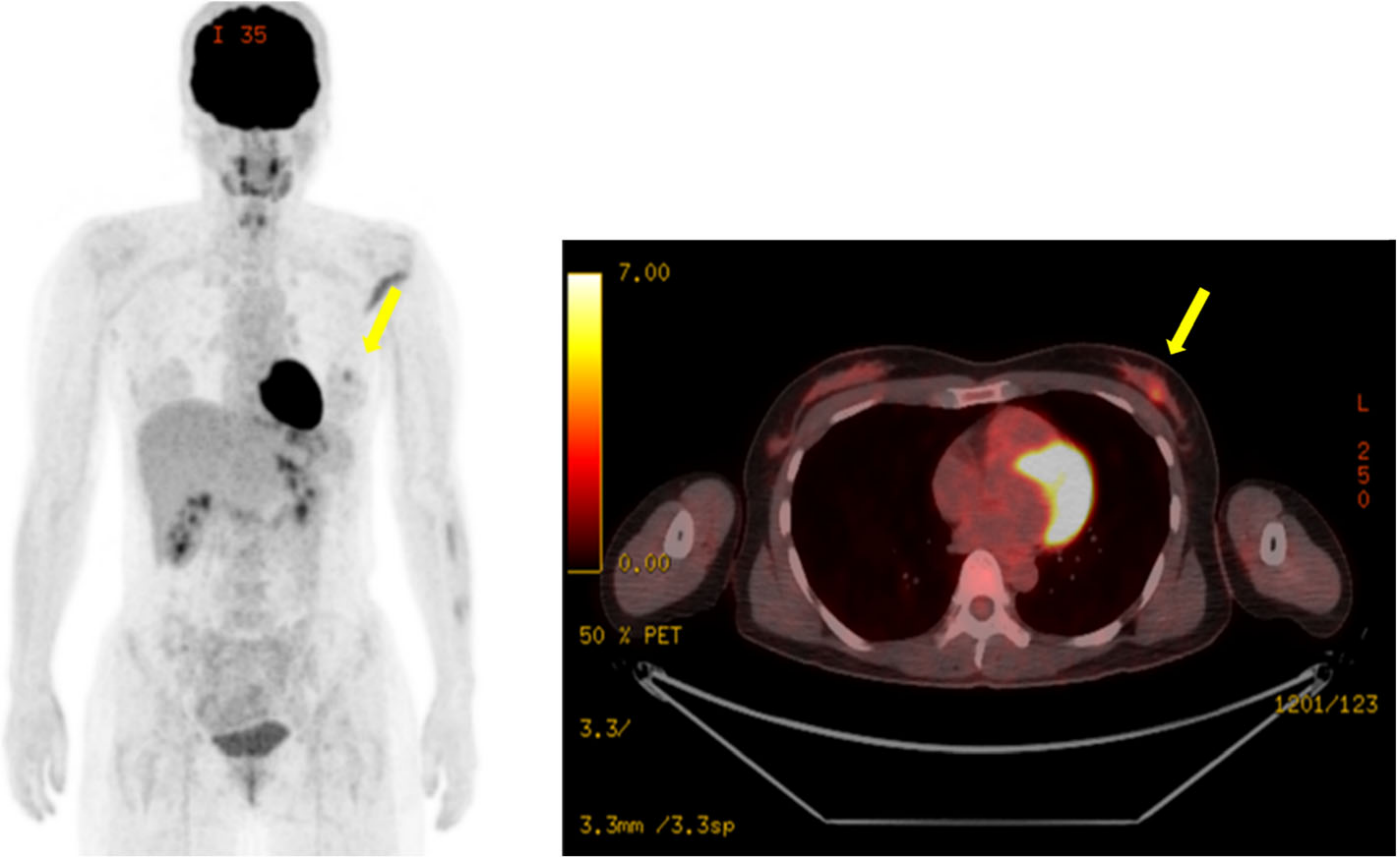
Whole-body fluorodeoxyglucose (FDG) positron emission tomography-computed tomography (PET-CT). Whole-body FDG PET-CT images showing a mass in the left C area with a maximum standardized uptake value of 3.54 (yellow arrows). Other abnormal accumulations were not detected in other axillary lymph nodes, lungs, liver, and bones

A 47-year-old Japanese woman presented with no subjective symptoms. Mammography revealed a clustered pleomorphic calcification in the left C region (Fig. 1a–d). She was referred to our hospital for further examinations. Her medical history stated that her mother had bilateral breast and ovarian cancers. Physical examination did not reveal a mass and papillary secretion in the breasts. The serum levels of the tumor markers carcinoembryonic antigen (CEA), CA15-3, NCC-ST-439, and BCA225 were normal. Ultrasonography identified a solid (13 mm × 12 mm × 7 mm), irregularly shaped low echo mass with a clear boundary in the left CD region and a high echo spot in the interior (Fig. 2). The findings of mammography and ultrasonography comprehensively classified the tumor as category 4, according to Breast Imaging Reporting and Data System (BI-RADS) 5th edition. Contrast-enhanced magnetic resonance imaging (MRI) revealed a lesion (14 mm × 11 mm × 12 mm) with clear margins in the left C region, confined to the same area (Fig. 3). Analysis of a core needle biopsy of this mass led to a diagnosis of ductal carcinoma in situ (DCIS), nuclear grade (NG) = 2, which was negative for the expression of estrogen receptor (ER), progesterone receptor (PgR), and human epidermal receptor 2 (HER2). The Ki 67 labeling index was 32.4%. Positron emission tomography-computed tomography (PET-CT) revealed a breast tumor with a maximum standardized fluorodeoxyglucose (FDG) uptake value of 3.54 in the left C region. However, we did not detect an abnormal accumulation of FDG that would indicate metastasis to the axillary lymph nodes, lungs, liver, or bones (Fig. 4).

**Fig. 5:**
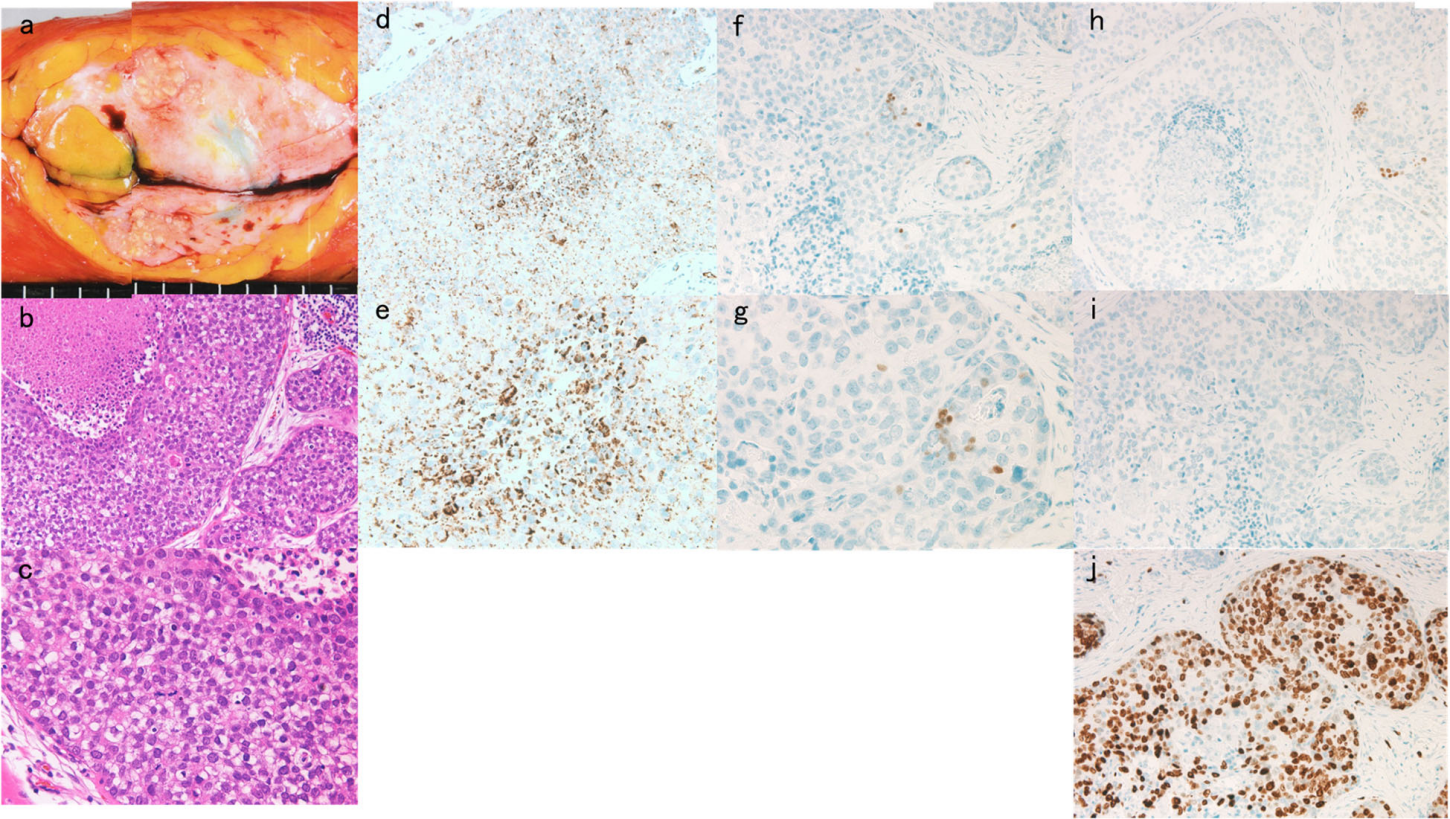
Macroscopic and pathological findings. **a** Macroscopic analysis showing relatively clear boundaries. A localized yellowish, white mass was observed, and continuity between the skin and papilla was not evident. **b** Histopathological analysis revealed carcinoma in situ as the dominant lesion. Hematoxylin and eosin staining revealed cytoplasmic clear and minute vacuolar structures of numerous tumor cells, indicating sebaceous differentiation. **c** Higher magnification of **b**. **d** Most tumor cells (50–90%) were adipophilin-positive. **e** Higher magnification of **d**. **f**–**i** Tumor cells did not detectably express estrogen receptor (ER), **f** progesterone receptor (PgR) (h), or human epidermal receptor 2 (HER2) (**i**). **g** Higher magnification of **f**. **j** The Ki 67 labeling index was 32.4%

Although hereditary breast and ovarian cancer syndrome (HBOC) could be considered a possibility because of the patient’s age and family history, she did not desire genetic counseling or *BRCA* testing. Upon the patient’s request, we performed total left mastectomy and sentinel lymph node biopsy (SLNB). SLNB was negative for metastasis and therefore axillary lymph node dissection was omitted. Macroscopic findings included a relative clear boundary and a yellowish white mass in the left C region, and continuity with the skin and papilla was not observed (Fig. 5a). Histopathology revealed stromal microinvasion (≤ 1-mm diameter) with predominant CIS. Numerous cells exhibited a clear and minute vacuolar cytoplasm, suggesting sebaceous differentiation (Fig. 5b, c). Immunohistochemical analysis revealed that 50 to 90% of the tumor cells expressed adipophilin (Fig. 5d, e), leading to a diagnosis of SC of the breast. The CIS lesions comprised tumor cells with similar phenotypes, and comedo necrosis was confirmed. Further tests did not detect venous or lymphatic invasion, NG = 3, or expression of ER-, PgR-, or HER2. The Ki 67 labeling index was 90% (Fig. 5f–j). Lymph node metastasis was not observed. Together, these findings were consistent with pathological T1mi, N0, M0 Stage I. The lesion was mainly CIS with minimal stromal invasion, and we therefore did not offer the patient chemotherapy. Metastasis or recurrence was not observed 16 months after surgery.

## Conclusions

SC is characterized by lobular forms or nests of tumor cells that exhibit sebaceous differentiation, which distinguishes SC from invasive ductal carcinoma (IDC) [11]. The World Health Organization classifies SC of the breast as primary breast carcinoma if at least half the constituent cells exhibit sebaceous differentiation and are not derived from cutaneous adnexal sebaceous glands as indicated by histopathological analysis [12]. Our present patient’s tumor originated deep within the mammary gland and was obviously separated from the adjoining skin. Further, lesions were present in the duct, which is consistent with our diagnosis of SC of the breast.

Published studies of SC of the breast do not establish its clinical significance. Thus, treatment typically follows that administered to patients with IDC.

Differential diseases of SC of the breast include histologically distinct glycogen-rich clear cell carcinomas and lipid-rich carcinomas. Glycogen-rich clear cell carcinomas typically become periodic acid-Shiff (PAS)-positive and therefore can be distinguished from SC cells because they do not produce glycogen [13]. Lipid-rich carcinoma is diagnosed according to the presence of tumor cells with a clear cytoplasm comprising at least 90% lipid-rich vacuoles [12]. Compared with SC of the breast, in which finely vacuolated cells grow into a compact lobulated solid form, the pattern of infiltration of lipid-rich carcinoma is similar to that of typical IDC and with much less conspicuous cellular vacuolization [14]. Thus, diagnosis is achieved through the demonstration of intracytoplasmic lipids detected using Oil Red O or Sudan Black B, or through immunohistochemical detection of the expression of adipophilin [15]. Because of its low incidence, there are no defined immunohistochemical features specific to SC of the breast, except for cytoplasmic lipids. Here, we found numerous clear cells with microvacuolar structures that we suspected to represent sebaceous differentiation. Further, 50–90% of the tumor cells were adipophilin-positive, which led to our diagnosis of SC of the breast.

Varga et al. [13], Ohara et al. [2], and Numoto et al. [16] propose hypotheses that explain the origin of SC of the breast, respectively, as follows: (a) The mammary glands of patients with breast cancer may exhibit features of sebaceous glands because these glands share the same origin. (b) SC of the breast develops from progenitor cells capable of differentiation into the sebaceous glands within the epithelium. (c) Sebaceous glands are ectopically present in the mammary gland.. If SC of the breast occurs in the absence of other breast cancer pathology, as is the case here, the possibility of scenarios b or c seems plausible.

A search of PubMed using the keywords “sebaceous carcinoma AND breast,” returned 16 case reports (Table 1) [4, 13, 14, 16–24]. These results support our assertion that the present report is the first to identify a patient with pT1mi, N0, M0, Stage I SC of the breast with predominant CIS. As mentioned above, ocular SC is infrequently associated with CIS. Moreover, evidence indicates that CIS is rarely associated with extraocular SC, although extraocular SC occurs in the head and neck and upper arm [7–10]. We identified a fatal case in which metastasis occurred over a relatively short period of 28 months after treatment [14], a case where local recurrence occurred 10 months after partial mastectomy [22], and a case in which skin and bone metastasis occurred 96 months after treatment [13]. Further, we identified a patient who experienced disease-free survival for 75 months after surgery [14]. However, the aspects of our present unique case do not allow us to offer a prognosis. Our current patient was not administered postoperative chemotherapy, and we will therefore conduct a careful follow-up.

**Table 1:** Survey of the literature describing the clinical and pathological features of sebaceous carcinoma of the breast

Authors	Year	Age	Sex	Surgery^†^		Pathological Findings															
				Breast	Axillary node	ER	PgR	AR	HER2	Subtype	NG	Ki67	IDC component	Adipophilin	Oil red O	Sudan Black B	pT	pN	M	Stage^‡^	Prognosis
Prescott RJ	1992	74	F	Bt	Ax								+		+		2	0	X	X	AWNED, 6 months
Mazzella FM	1995	55	M	Bt	Ax	+	+			Luminal	2	11	+		+		2	0	X	X	AWNED, 10 months
Tavassoli FA	1999	46	F			−	+	−		Luminal		20					3	0	X	X	NA
Propeck PA	2000	46	F	Tm→Bp													1	X	X	X	AWEND, 6 months
Varga Z	2000	45	F	Bt	Ax	+	+	−	−	Luminal		16	+				2	0	0	IIA→IV	AWD, 96 months
Hisaoka M	2006	63	F	Bt	Ax	+	+	−	−	Luminal		38			+		2	1	X	X	NA
Numoto S	2007	49	F	Tm		+	+	+	−	Luminal		15	+				1	X	X	X	NA
Murakami A	2009	50	F	Bt	Ax	−	−	±	+	HER2		30	+	+			1	1	0	IIA	AWNED, 24 months
Ramljak V	2010	85	F	Bp→Bt		−	−		−	TN		25					2	X	X	X	AWD, 10 months
Kiyohara T	2013	76	F	Bp	SLNB												3	0	0	IIIA	AWNED, 32 months
Svajdler M	2015	65	F	Bp	SLNB→Ax	+	+		−	Luminal	3	30	+				1	1	0	IIA	AWNED, 27 months
Svajdler M	2015	61	F	Bt	Ax	−	−		−	TN	3	80					2	1	0	IIA→IV	DOD, 28 months
Svajdler M	2015	66	F	Bt	Ax	+	−		−	Luminal	2	5					2	1	0	IIA→IV	AWD, 70 months
Svajdler M	2015	25	F	Bp	Ax	+	+		−	Luminal	3						X	0	X	X	AWNED, 75 months
Yamamoto Y	2017	80	F	Bp	SLNB	−	−	−	−	TN	1			+		+	2	0	0	IIA	AWNED, 16 months
Ohno K	2019	47	F	Bt	SLNB	−	−		−	TN	3	90		+			1mi	0	0	I	AWNED, 16 months

*Abbreviations*: *AR* androgen receptor, *Ax* axillary dissection, *AWD* alive with disease, *AWNED* alive with no evidence of disease, *Bp* partial mastectomy, *Bt* total mastectomy, *DOD* died of disease, *ER* estrogen receptor, *F* female, *HER2* human epidermal growth factor receptor type 2, *IDC* invasive ductal carcinoma, *M* male, *NA* not available, *NG* nuclear grade, *PgR* progesterone receptor, *SLNB* sentinel lymph node biopsy, *Tm* tumorectomy, *TN* triple negative

^†^Change in surgery is indicated by an arrow

^‡^According to International Union Against Cancer TNM Classification of Malignant Tumors (8th Edition). Disease progression indicated by an arrow

In conclusion, here, we encountered a rare case of SC of the breast with predominant CIS. Although our findings contribute to our understanding of the pathogenesis, progression, and prognosis of SC of the breast, further studies of additional cases are required.

## Data Availability

The dataset supporting the conclusions of this article are available in the manuscript.

## Abbreviations

AR: Androgen receptor
BI-RADS: Breast imaging reporting and data system
CEA: Carcinoembryonic antigen
CE-MRI: Contrast-enhanced magnetic resonance imaging
CIS: Carcinoma in situ
DCIS: Ductal carcinoma in situ
ER: Estrogen receptor
HBOC: Hereditary breast and ovarian cancer syndrome
HER2: Human epidermal receptor 2
IDC: Invasive ductal carcinoma
MRI: Magnetic resonance imaging
NG: Nuclear grade
PAS: Periodic acid-Shiff
PET-CT: Positron emission tomography-computed tomography
PgR: Progesterone receptor
SC: Sebaceous carcinoma
SLNB: Sentinel lymph node biopsy
